# A longitudinal sampling study of transcriptomic and epigenetic profiles in patients with thrombocytopenia syndrome

**DOI:** 10.1038/s41467-021-25804-z

**Published:** 2021-09-24

**Authors:** Yafen Wang, Shaoqing Han, Ruoxi Ran, Anling Li, Huanyu Liu, Mingjun Liu, Yongwei Duan, Xiong Zhang, Zhigang Zhao, Shihui Song, Xiaocheng Weng, Song-Mei Liu, Xiang Zhou

**Affiliations:** 1 College of Chemistry and Molecular Sciences, Wuhan University, Wuhan, China; 2 Department of Clinical Laboratory, Center for Gene Diagnosis, and Program of Clinical Laboratory Medicine, Zhongnan Hospital of Wuhan University, Wuhan, China; 3 Department of Obstetrics and Gynecology, Reproductive Medicine Center, Zhongnan Hospital of Wuhan University, Wuhan, China; 4 Department of Emergency, Zhongnan Hospital of Wuhan University, Wuhan University, Wuhan, China; 5 Department of Infectious Disease, Zhongnan Hospital of Wuhan University, Wuhan University, Wuhan, China

**Keywords:** Epigenetics, Virology, Virus-host interactions

## Abstract

Severe fever with thrombocytopenia syndrome (SFTS) is a novel tick-borne infectious disease caused by a new type of SFTS virus (SFTSV). Here, a longitudinal sampling study is conducted to explore the differences in transcript levels after SFTSV infection, and to characterize the transcriptomic and epigenetic profiles of hospitalized patients. The results reveal significant changes in the mRNA expression of certain genes from onset to recovery. Moreover, m^6^A-seq reveals that certain genes related with immune regulation may be regulated by m^6^A. Besides the routine tests such as platelet counts, serum ALT and AST levels testing, distinct changes in myocardial enzymes, coagulation function, and inflammation are well correlated with the clinical data and sequencing data, suggesting that clinical practitioners should monitor the above indicators to track disease progression and guide personalized treatment. In this study, the transcript changes and RNA modification may lend a fresh perspective to our understanding of the SFTSV and play a significant role in the discovery of drugs for effective treatment of this disease.

## Abstract (web summary)

Severe fever with thrombocytopenia syndrome (SFTS) is an emerging hemorrhagic fever caused by tick-borne SFTS virus. Here, Wang et al. characterize transcriptomic and epigenetic changes in infected patients and correlate them with clinical parameters to improve the understanding of disease progression.

## Introduction

In 2009, an emerging severe fever with thrombocytopenia syndrome (SFTS) caused by the SFTS virus (SFTSV) was identified for the first time in China^1–3^ and was subsequently found to be widely distributed in 23 provinces of China^4^ and other Asian countries and to have a mortality rate ranging between 12 and 50%^5–8^. Heartland virus, another phlebovirus that is genetically closely related to SFTSV, was reported in the USA in 2012^9^. Due to its high mortality rate and the possibility of an outbreak of as a global pandemic, SFTS was declared one of the top ten priority infectious diseases by the WHO in 2017^8^.

SFTSV is a tick-borne phlebovirus belonging to the genus Phlebovirus in the family Phenuiviridae (order Bunyavirales)^1,10^. Farmers in endemic areas (living in wooded or hilly regions and working in fields) are more likely to be exposed to ticks and hence more vulnerable to the SFTSV infection^11^. Contact with blood or excretions from infected patients is another route of SFTSV transmission^12^. The progression of SFTS includes four typical stages: incubation, fever, organ failure, and convalescence or death. The incubation period after SFTSV infection is generally proximately 5–14 days. The acute onset of the fever stage (temperature ranging from 38 to 41 °C) lasts for 5–11 days and also characterized by influenza-like symptoms such as headache, myalgia, vomiting, diarrhea, poor appetite, and fatigue. Multiple organ failure is observed in severe cases. The laboratory results of SFTS patients are characterized by reduced leukocytes and platelets, increased alanine and aspartate aminotransferases, and proteinuria^12,13^. To date, several vaccines have been developed and found to protect against SFTSV infections in ferrets^10,14–16^. However, no effective therapy or vaccine is yet available for clinical use. As no specific antiviral drugs have been developed for the treatment of SFTSV infection, understanding the pathogenic mechanism of the SFTSV is crucial for developing appropriate antivirus therapeutic strategies.

The genome of SFTSV consists of three negatively-stranded RNA segments including the small (S), medium (M), and large (L) segments, which contain 1744, 3378, and 6368 nucleotides, respectively^17,18^. Notably, the S segment encodes the nonstructural protein (NSs) and the nucleoprotein (NP) in the reverse direction protecting the genomic RNA from degradation by exogenous nucleases or the immune systems in the host cells and play a crucial role in the replication of SFTSV. Normally, S segment PCR amplification is used to confirm the diagnosis of SFTSV infection^17,18^. Moreover, the RNA-dependent RNA polymerase (RdRp), encoded by the L segment, has been found to be vital for viral RNA transcription and replication. The M segment-encoded glycoprotein (GP) has been found to be involved in virus assembly, the formation of virus particles, and attachment to host cells.

Although the architecture of SFTSV has been determined, the associated transcriptional regulatory alterations, specifically, the epigenetic landscape of the virus and the host cell, remain poorly characterized. Posttranscriptional modifications, such as N^6^-methyladenosine (m^6^A), are known to regulate the life cycles of certain viruses, such as human immunodeficiency virus (HIV), hepatitis B virus (HBV), and even human coronavirus, and to play critical roles in the transcriptional responses associated with viral infection^19–21^. To date, very few studies have focused on the transcriptomic analysis of patients with SFTSV, which is important to thoroughly understand the life cycle and pathogenicity of this virus.

In this work, to investigate the pathogenetic mechanism of SFTSV infection, we analyze the chronological changes in laboratory findings and perform transcriptomic and epitranscriptomic analyses of blood samples from patients with SFTSV infection that are obtained at multiple timepoints from admission to discharge. The differentially expressed genes identified through RNA-seq are consistent with the symptoms exhibited at the corresponding stages of SFTSV infection. These findings can contribute to the understanding of the pathophysiology of SFTSV and thus pave the way for a breakthrough in therapy. The integration of clinical features with transcriptomic and epigenetic data provides researchers with fresh insights into human cellular responses to SFTSV infection.

## Results

### Clinical features of patients with SFTSV infection

The serum SFSTV viral loads in all patients ranged from 34 to 9.49 × 10^8^ IU/mL, and platelet counts varied from 4.0 × 10^9^/L to 88.0 × 10^9^/L. As shown in Table 1 and Supplementary Fig. 1, the baseline laboratory findings included thrombocytopenia, leukopenia, lymphocytosis, erythropenia, and elevated alanine aminotransferase (ALT), aspartate transaminase (AST), blood urea nitrogen (BUN), and creatinine, i.e., white blood cell (WBC) counts declined in 36.6% of the cases, serum ALT increased in 78.0% of the cases, and all cases had reduced platelet counts and elevated AST. Given that 11 out of 41 patients died of SFTSV, we compared the clinical features between the recovered patients and the deceased patients. The deceased patients showed: (1) a shorter duration of SFTS (*p* < 0.001), defined as days from the onset of symptoms to clinical outcome, (2) a higher viral load and more rapid disease progression, and (3) lower RBC counts along with higher levels of serum ALT, AST, BUN, creatinine, and activated partial thromboplastin time (APTT), suggesting that multiple organ damage is common in severe cases (Table 1 and Supplementary Fig. 1).

**Table 1:** Baseline characteristics in all patients infected with SFTSV.

Variables	Recovered patients (*n* = 30)	Deceased patients (*n* = 11)	*P* value
Age, years	63.6 (56.2–71.1)	65.4 (62.7–71.3)	0.494
Males, %	18 (58.1%)	6 (60.0%)	0.914
Duration, days^a^	19.0 (16.0–24.3)	10.0 (8.0–11.0)	1.80 × 10^−5^
SFTSV, IU/mL^b^	1.66 × 10^4^ (5.60 × 10^2^−2.28 × 10^5^)	3.67 × 10^6^ (4.01 × 10^5^−3.83 × 10^7^)	4.961 × 10^−4^
*Complete blood count*			
WBC, ×10^9^/ L	3.46 (1.90–4.35)	2.75 (1.68–7.17)	0.976
Neutrophil, ×10^9^/L	1.87 (1.20–3.81)	1.15 (0.73–5.15)	0.564
Lymphocyte, ×10^9^/L	0.60 (0.46–1.04)	0.76 (0.50–1.67)	0.387
RBC, ×10^12^/L	4.12 (3.88–4.47)	3.61 (3.42–4.26)	0.029
Hemoglobin, g/L	129.0 (118.6–140.0)	120.0 (104.8–141.0)	0.274
Platelet, ×10^9^/L	43.0 (22.0–55.0)	35.0 (14.0–45.8)	0.261
CRP, mg/L	3.60 (2.19–18.30)	8.96 (6.07–11.84)	0.351
*Coagulation*			
PT, s	11.60 (10.60–12.20)	12.10 (11.63–12.70)	0.164
APTT, s	37.20 (33.00–44.40)	63.50 (47.40–66.75)	2.368 × 10^−4^
*Liver function*			
ALT, U/L	88.0 (47.0–147.0)	142.00 (108.5–191.5)	0.033
AST, U/L	221.0 (108.0–486.0)	825.00 (459.0–1299.5)	1.132 × 10^−3^
TBIL, μmol/L	10.20 (7.80–13.40)	10.30 (9.40–22.80)	0.315
*Renal function*			
BUN, mmol/L	5.04 (3.70–6.72)	7.95 (4.83–12.55)	0.022
Creatinine, μmol/L	68.0 (62.60–84.40)	90.00 (70.50–145.80)	0.041

Data are median (IQR) or *n* (%). *P* values were calculated by two-sided Mann–Whitney *U* test or *χ*² test.

*WBC* white blood cell, *RBC* red blood cell, C*RP* C-reactive protein, *PT* prothrombin time, *APTT* activated partial thromboplastin time, *ALT* alanine aminotransferase, *AST* aspartate aminotransferase, *TBIL* total bilirubin, *BUN* blood urea nitrogen.

^a^Duration was defined as days from onset to outcomes.

^b^IU indicates 50% tissue culture infective dose (TCID50), 10x TCID50/mL = 0.496 × 10^x+3^ copies/mL. Statistic analysis was performed with log10-transformation of SFTSV viral load.

**Fig. 1:**
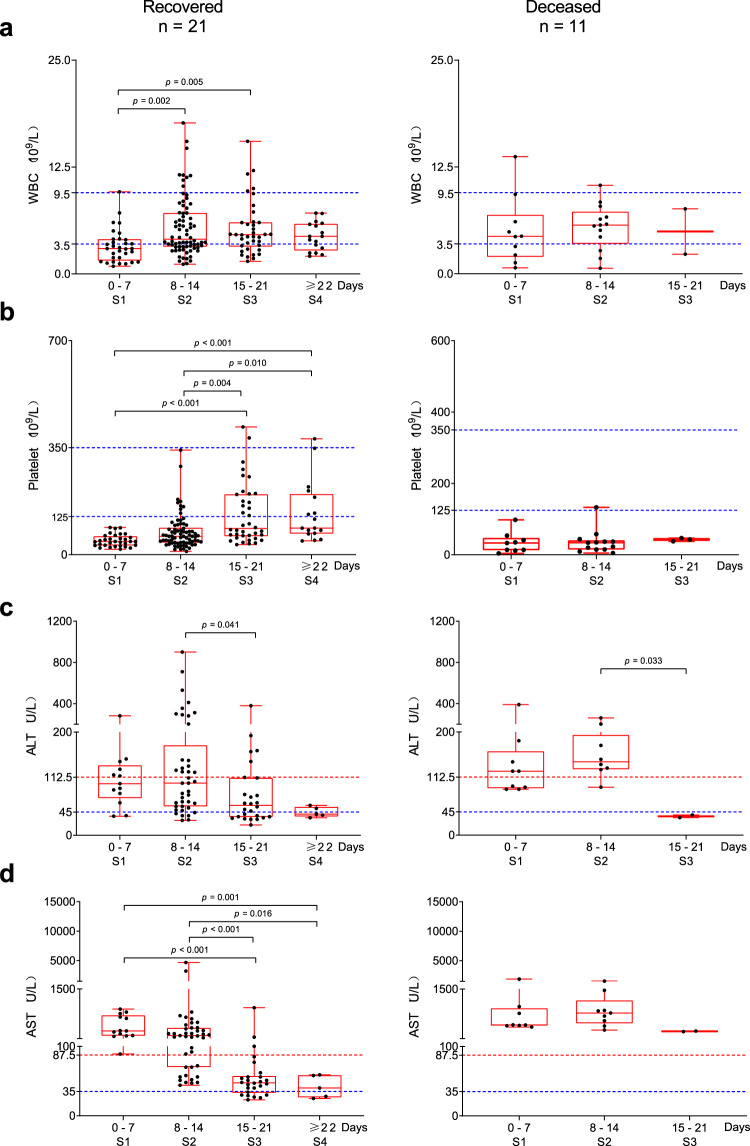
Chronological changes of laboratory findings at different stages in patients with SFTS. The progress of the SFTS, from the onset of symptoms to outcomes, is classified into four stages. Comparison of WBC counts (**a**), platelet counts (**b**), serum ALT (**c**) and AST (**d**) among groups are shown in the left panel (recovered patients, *n* = 21) and the right panel (deceased patients, *n* = 11), respectively. The dashed blue lines represent the clinical normal ranges of WBC (3.5–9.5 × 10^9^/L), platelet (125–350 × 10^9^/L), ALT (0–45 U/L), AST (0–35 U/L). The dashed red lines represent 2.5-fold of the upper limit of the normal ranges for ALT and AST, which indicates requiring supportive treatment for liver function. Each dot represents the result of a single test. Stage: S1 (Fever stage); S2 (Deterioration/Organ failure); S3 (Improving); S4 (Convalescence). A two-sided Mann–Whitney *U* test was used to assess the difference between groups and the *p* values are shown on the graph. In the presence of significant interactions, corrected pairwise comparisons were performed using a Bonferroni correction. Top line, maxima; bottom line, minima; center line, median; box limits, upper and lower quartiles; whiskers, 1.5× interquartile range; points, values.

To examine the dynamic changes during the progression of SFTS, laboratory and clinical data were collected from the records of all deceased patients (*n* = 11), and recovered patients who had blood cell counts and liver function testing more than 5 times during their clinical course (*n* = 21). Based on clinical presentations, the data were then classified into 4 groups according to SFTS stage from the onset of symptoms to outcomes: fever stage (0–7 days), deterioration/organ failure (8–14 days), improvement (15–21 days)/death (15–21 days), and convalescence (≥22 days). The general pattern and differences among groups were evaluated, and the results are shown as follows (Fig. 1, Supplementary Fig. 1): (1) WBC counts were found to be the lowest at the fever stage and were below the normal range in more than half of the cases. An abnormal increase of WBC counts with the disease deterioration and/or organ failure, which could indicate other pathogenic infections (Fig. 1a). (2) The platelet counts were also lowest at the fever stage. With disease improvement, the platelet crises were well controlled, and platelet counts significantly increased. Platelet crisis was defined as a platelet count less than 30 × 10^9^/L (Fig. 1b). (3) During the improving stage, serum ALT levels dropped to 2.5-fold of the upper limit of the normal range in 80.8% of recovered patients, indicating the restoration of liver function (Fig. 1c). (4) Serum AST levels were dramatically elevated during the fever stage and significantly declined during the improving stage and convalescence stage in the recovered patients (Fig. 1d).

Taken together, in addition to abnormal WBC counts, all deceased patients showed abnormal platelet counts and serum ALT and AST levels. Of note, AST levels were >10-fold higher than the upper limit of the normal range, and platelet counts were persistently below 60 × 10^9^/L (Fig. 1, Supplementary Fig. 1), demonstrating that dramatic increase in AST and a decrease in platelet counts indicated poor prognosis.

### SFTSV infection alters the transcriptome of SFTS patients

**Fig. 2:**
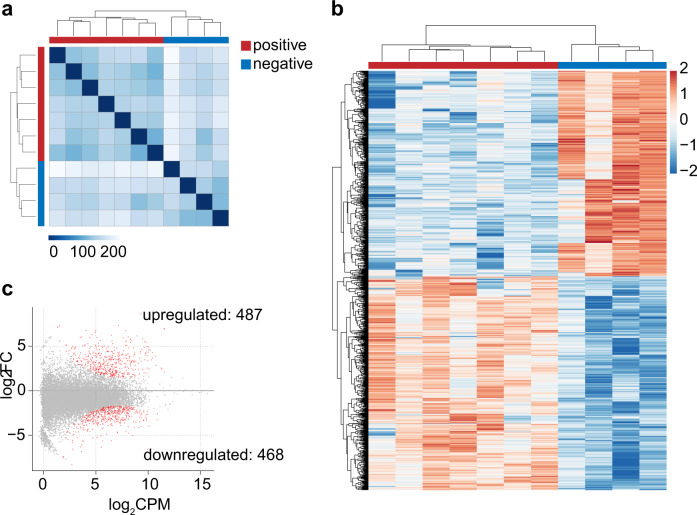
SFTSV infection alters the transcriptome. RNA-seq was performed on RNA isolated from the white blood cell from 7 positive samples and 4 recovery-negative group (negative samples from recovery patients). **a** Hierachical clustering of samples according to global gene expression demonstrates positive samples (red) cluster together whereas negative samples (blue) segregate together. Detailed information of the patients are provided in Supplementary Table 1. **b** Heat map of the z-scores for 955 significantly differentially expressed genes identified using RNA-seq (*P* < 0.001, exact test, likelihood ratio test, and quasi-likehood *F* test) shows that genes distinguish the SFTSV-positive samples from the SFTSV-negative samples. Red represents increased relative expression, and blue represents decreased relative expression. **c** Differen**c**e gene-expression in SFTSV virus (positive) and SFTSV virus (negative) with *P* < 0.001, exact test, likelihood ratio test, and quasi-likelihood *F* test. Red points indicate increase or decrease significantly while gray means no significant difference in negative and positive samples. FC, fold change; CPM, read count per million.

To better understand the pathogenetic mechanism of the SFTSV infection, exploring some strong clues for the targeted treatment of SFTS, we performed RNA-seq to reveal the differentially expressed genes of the patients from the period of SFTSV infection to the recovery stage. Here, 7 SFTSV-positive samples and 4 SFTSV-negative samples from the SFTS patients were used for RNA-seq analysis. First, the RNA-seq data of these 11 samples were analyzed for the differentially expressed genes. As shown in Fig. 2a, the SFTSV-positive samples clustered together, nonetheless, separately from the SFTSV-negative samples in the hierarchical clustering of transcriptome-wide gene expression, revealing systemic alterations in RNA expression. The differential gene expression analysis revealed 955 differentially expressed genes between the SFTSV-positive samples and the SFTSV-negative samples (*P* < 0.001, exact test, likelihood ratio test, and quasi-likelihood F test) (Fig. 2b). Of these, 468 genes were downregulated, while 487 genes were upregulated upon SFTSV infection (Fig. 2c).

In addition, some blood samples covering different stages of infection were collected from three patients. The change patterns of SFTS viral loads and platelet counts of these three patients were summarized (Supplementary Fig. 2). As expected, the platelet counts returned to the normal ranges gradually along with the inhibition of the replication of SFTSV, although the viral load rebounded occasionally during the treatment.

**Fig. 3:**
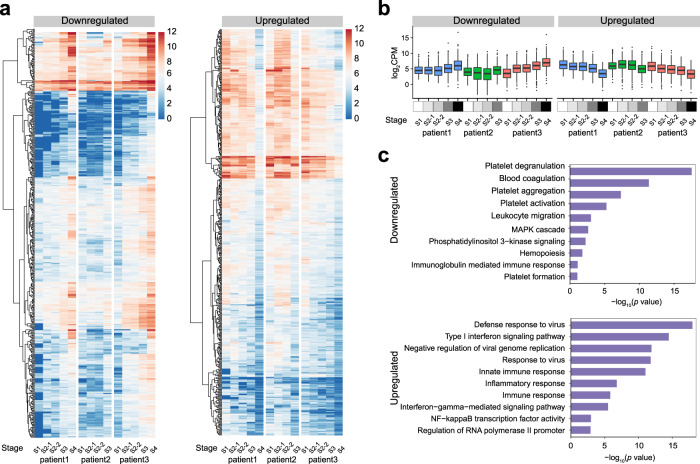
Differential gene landscapes and pathway analysis at different stages of SFTSV infection. **a**, **b** Three patients with covering different stages of infection. **a** Clustered heat map showing significant differential expression of 955 genes identified by RNA-seq at different stages of SFTSV infection. Stage: S1 (Fever stage); S2 (Deterioration/Organ failure), S2-1 represents the early stage of S2 and S2-2 represents the later stage of S2; S3 (Improving); S4 (Convalescence). **b** Box plots showing the 955 genes CPM levels of three patients at different stages. The left and right dotted lines show downregulation (the SFTSV-positive sample was downregulated compared with the SFTSV-negative sample, *n* = 468 genes) and upregulation (the SFTSV-positive sample was upregulated compared with the SFTSV-negative sample, *n* = 487 genes) of gene expression after SFTSV infection, respectively. Center line, median; box limits, upper and lower quartiles; whiskers, 1.5× interquartile range; points, outliers. **c** GO analysis of the human transcripts containing differential expression genes upon SFTSV infection using DAVID bioinformatics database (the *p* values are provided by DAVID online tool).

Among the samples from 3 patients with covering different stages of infection, some differentially expressed genes identified in the RNA-seq of 11 samples (7 SFTSV-positive specimens and 4 SFTSV-negative specimens) were found to correlate to the different disease stages (Fig. 3a, b). For example, the expression levels of these differential genes were higher in the negative samples than in the positive samples, and their expression levels increased gradually as the patients recovered. In contrast, the expression of those genes with high expression in the positive samples decreased gradually as the patients recovered.

To acquire better knowledge about the biological functions of these differentially expressed genes, clusterProfiler identification (Gene Ontology, GO) was applied to annotate the pathways of these genes. The GO analysis of genes from each cluster revealed that the downregulated genes tended to execute functions, including platelet degranulation (*P* = 2.59 × 10^−18^), blood coagulation (*P* = 3.88 × 10^−12^), platelet aggregation (*P* = 4.12 × 10^−8^), and platelet activation (*P* = 5.03 × 10^−6^), presumably providing energy and material support for the basic requirements of blood supplementation (Fig. 3c). Consistent with the functions of these differential genes, all the patients indicated dramatically decreased platelet counts relative to the normal range (125–350 × 10^9^/L) (Table 1), and the average platelet counts in the recovered and deceased patients were found to be approximately 43.0 × 10^9^/L and 35.0 × 10^9^/L, respectively. In addition, significantly prolonged APTT compared to the normal range (25.1–36.5 s) was observed in both the recovering (37.2 s) and the deceased patients (63.5 s), implying that blood coagulation was impaired in the patients. The upregulated genes on the other hand, fell into the category of defense mechanism against the virus (*P* = 9.03 × 10^−19^), negative regulation of the viral genome replication (*P* = 1.05 × 10^−12^), response to the virus (*P* = 1.4 × 10^−12^), innate immune response (*P* = 8.26 × 10^−12^), and inflammatory response (*P* = 1.43 × 10^−7^), implying that these biological processes underlie the immunoreaction. These biological processes were indeed closely associated with the clinical manifestations of the patients’ response to SFTSV infection, such as sharp decreases in platelet counts, leukopenia, and lymphocytosis. Gene Ontology analysis of the genes that were differentially expressed in the positive vs negative samples identified pathways associated with leukocyte migration, regulation of blood coagulation, and platelet formation as enriched in patients infected with SFTSV.

As one example, the platelet glycoprotein Ib alpha chain is a platelet surface membrane protein encoded by the *GP1BA* gene, which could influence the risk of juvenile idiopathic arthritis and atherothrombosis mediated platelet counts^22–24^. Filamin A (*FLNA*) is the most abundant and widely distributed member in the filamin protein family and was recently observed to be involved in platelet biology, including reproduction and activation^25–27^. Integrin β3 was encoded by the *ITGB3* gene and forms platelet glycoprotein (GP) IIb/IIIa, which acts as the principal platelet receptor for fibrinogen^28^. In the platelet-associated pathways, the expression levels of *GP1BA, FLNA*, and *ITGB3* were found to be downregulated in the patients with positive serum SFTSV tests. These features remained consistent with the decline in platelet counts and the prolonged APTT.

In addition, interferon-induced transmembrane proteins (IFITMs), belonging to the Dispanin/CD225 family, are comprehensive transmembrane proteins induced by interferon. IFITMs exhibit active roles in the replication and invasion of several types of viruses, such as SARS-CoV-2 and influenza A virus^29–33^. Upregulation of the *IFITM1* and *IFITM3* was observed in the SFTSV-positive samples compared to the negative samples, implying that these IFITM proteins may effectively suppress the host cell invasion of SFTSV (Supplementary Fig. 3). The RNA-seq expression results for certain differentially expressed genes were also further validated by qPCR (Supplementary Fig. 4). The expression trend of these genes in positive and negative samples was consistent with the results of RNA-seq.

### Correlation analysis of RNA-seq data and clinical indicators

**Fig. 4:**
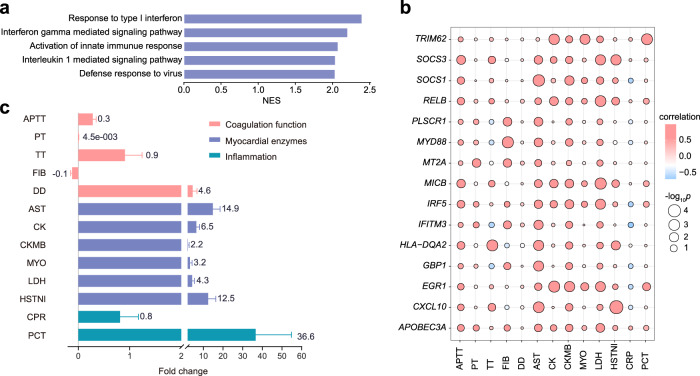
Correlation between clinical outcomes and sequencing data. **a** The core-enriched signaling pathways. Normalized enrichment score (NES suggested the results across the analyzed genes. **b** Dot plot showing the genes associated with the clinical indexes related to coagulation function, myocardial enzymes, and inflammation (Pearson correlation coefficient (*r*), and two-sided *t*-tests). **c** Fold changes of laboratory results from 41 patients infected with SFTSV at admission are shown on the graphs. The fold changes are calculated according to the following formula: Fold changes = (A−B)/B (A indicates the clinical testing results at admission, B represents the up or low limit of the normal ranges, *n* = 41 patients, error bars represent standard error of means (SEM)). LAPTT activated partial thromboplastin time, TT thrombin time, FIB fibrinogen, DD D-dimer, AST aspartate aminotransferase, CK creatine kinase, CKMB creatine kinase MB, MYO myoglobin, LDH lactate dehydrogenase, HSTNI hypersensitive troponin I, CRP C-reactive protein, PCT procalcitonin.

To explore the correlation between RNA-seq data and clinical outcomes, we first identified 125 differentially expressed genes across the deceased group, recovery-positive group (positive samples from recovering patients), recovery-negative group (negative samples from recovering patients), and healthy group (Supplementary Figs. 5, 6). These genes were mainly enriched in virus infection pathways and inflammation pathways, such as response to type I interferon, interferon-gamma-mediated signaling pathway, activation of innate immune response, interleukin 1 mediated signaling pathway, and defense response to virus (Fig. 4a). Pearson correlation analysis was used to identify clinical indicators that were highly correlated (absolute value of Pearson *r* ≥ 0.8) with the 125 differentially expressed genes and found that the genes were mainly related to myocardial enzymes, coagulation function, and inflammation (Fig. 4b). Further validation using the clinical data from 41 hospitalized patients in 2020 showed consistent significant changes in the following indicators compared with their corresponding normal ranges, i.e., D-dimer (DD) (fold change = 4.6), AST (fold change = 14.9), creatine kinase (CK) (fold change = 6.5), lactate dehydrogenase (LDH) (fold change = 4.3), hypersensitive troponin I (HSTNI) (fold change = 12.5), and procalcitonin (PCT) (fold change = 36.6). Collectively, these results suggest that clinical practitioners may include testing for myocardial enzymes, coagulation function, and inflammation to monitor disease progression and provide personalized treatment, in addition to evaluating blood cell count, liver function, and renal function.

Next, we performed an integrated analysis of immune and inflammatory cytokines. Interleukin-6 (IL-6) plays an important role in inflammation and autoimmune disease. The expression levels of the genes *IL6R*, *STAT3*, and *SOCS3*, which are induced by IL-6, were significantly increased in the SFTSV-positive samples (Supplementary Fig. 7). Similarly, the expression levels of B cell activation related genes (*TNFSF13B*, *IL4R*), T cell exhaustion related genes (*BATF*, *IRF4*, *CD274*) and type I interferon related genes (*MX1*, *IFITM1*, *IFIT2*) were also increased in the patients. All these important initiation signals for cytokines indicated that cytokine storms may be the critical factor leading to organ damage and even death during disease progression. We are hopeful that the suppression of cytokine storms will be effective and allow the recovery of patients infected by SFTSV.

### SFTSV infection affects m^6^A modifications in SFTS patients

Epigenetic modification is known to be involved in several important biological processes. N^6^-methyladenosine (m^6^A) is the most abundant internal mRNA modification and is a reversible epitranscriptomic marker. Alteration of m^6^A has been found to be closely associated with diseases including viral infections (HSV, HBV, HCV, HIV)^21,34,35^, acute myeloid leukemia (AML)^36^, diabetes^37^, lung cancer^38^, hepatocellular carcinoma^39^, and breast cancer^40^. Therefore, it was reasonable to presume that the distribution or modification levels of RNA m^6^A might also be dysregulated in patients infected with SFTSV. To examine whether there was a change in the m^6^A distribution or content in the patients with SFTSV infection, several of these samples were subjected to immunoprecipitation with an anti-m^6^A affinity purification antibody. Immunoprecipitation and the input of the control fragments were used to prepare the library, and parallel sequencing of the healthy donors served as the healthy controls. m^6^A-seq analysis was performed on SFTSV-positive blood samples from four patients, and blood samples from three groups of healthy controls (each group included seven healthy individuals).

**Fig. 5:**
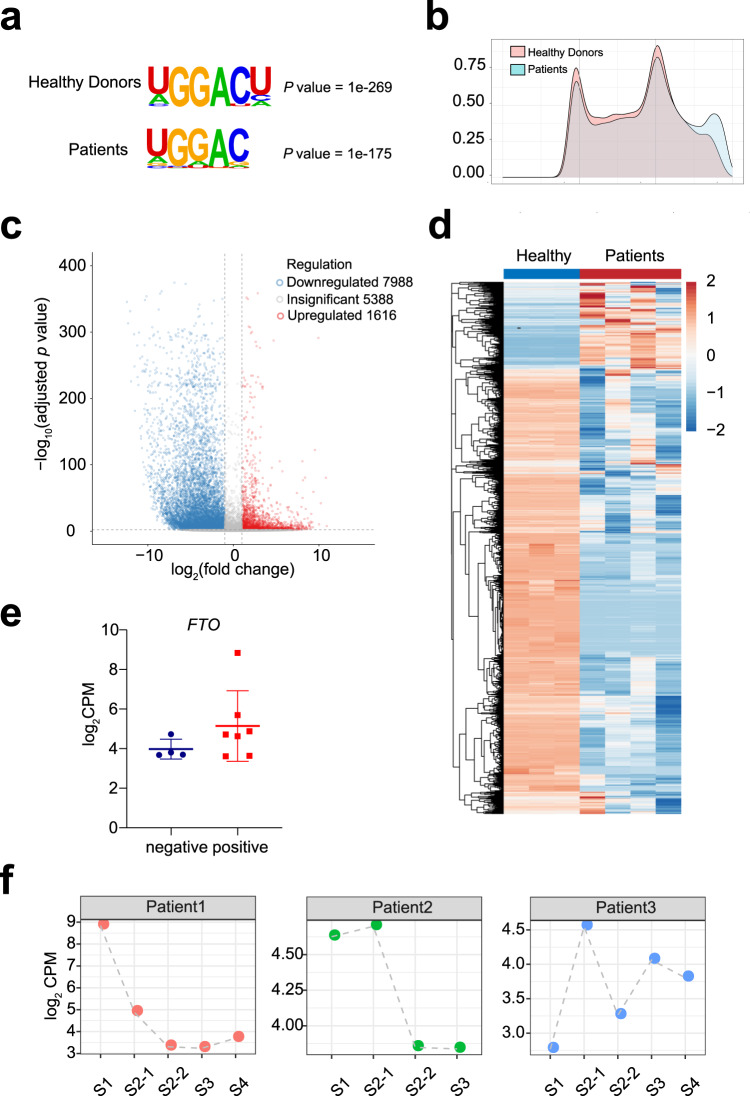
SFTSV infection affects m^6^A modification. **a** Sequence motifs enriched within m^6^A peaks identified by m^6^A-seq. **b** Metagene profile showing the distribution of m^6^A peaks across the length of transcripts composed of three transcript segments, i.e., 5′-untranslated region (5′-UTR), CDS, and 3′-untranslated region (3′-UTR), in SFTSV-infection samples. **c** Volcano plots showing the m^6^A enrichment in mRNAs of SFTSV-positive samples and healthy controls′ samples. Each dot represents a gene. The abundance of m^6^A with substantially increase (up) and decreased (down) enrichment are highlighted in red dots and blue dots, respectively (FDR-adjusted *p* value < 0.05). **d** Heat map of the peak log_2_ counts-per-million IP data of all differentially methylated genes. In the differential peaks of m^6^A, the majority of m^6^A abundance were downregulated in SFTSV-positive samples. **e** Each point represents the log_2_CPM of *FTO* value for either 4 negative or 7 positive samples (error bars are represented as mean values ± SD, this corresponds to the samples in Fig. 2, *p* value = 0.07, one-sided *t*-test). **f** The *FTO* CPM levels at different stages in the three patients (this corresponds to the samples in Fig. 3a, b).

Approximately 11,520 of the m^6^A peaks in the SFTSV-infected samples were also found in the healthy controls (Supplementary Fig. 8a). The sequence analysis of the m^6^A peaks revealed that the RRACH (R = A or G, H = A, T, or C) motif was highly enriched within the m^6^A peaks (Fig. 5a). The m^6^A modifications were predominantly distributed in the coding sequence (CDS), near the stop codons and the 3′ untranslated region (3′-UTR) (Fig. 5b and Supplementary Fig. 8b, c). The confluence of the m^6^A motif and the distribution indicated the high quality of the m^6^A-seq data. A comparison of the abundance of the m^6^A peaks between the patients and the healthy controls confirmed that the m^6^A modification level exhibited a significant decrease after SFTSV infection, with a total of 1616 m^6^A-containing genes upregulated and 7988 m^6^A contained genes downregulated (Fig. 5c). The heat map with the hierarchical clustering of samples based on the differentially methylated genes indicated a significant separation between the transcripts of the patients and the healthy controls (Fig. 5d). Gene Ontology term analysis illustrated that the genes with m^6^A modification changes were mainly enriched in immune response (*P* = 3.41 × 10^−16^), inflammatory response (*P* = 4.1 × 10^−15^), defense response to virus (*P* = 8.49 × 10^−11^), and viral transcription (*P* = 0.007) (Supplementary Fig. 9). These findings were consistent with the RNA-seq results, confirming that the changes in gene expression related to SFTSV infection may be regulated through an m^6^A-dependent pathway.

Of the 125 differentially expressed genes (death vs recovery-positive vs recovery-negative vs healthy), 112 genes exhibited differential m^6^A patterns between the positive samples and the healthy donors. Genes related to immune regulation and interferons, such as *ISG15*^41^, *TRIM26*^42^, and *IRF9*^43^, were highly expressed in the positive samples, and the abundance of m^6^A in these genes was lower than in the healthy controls. Fat mass and obesity-associated protein (FTO) play a vital role in m^6^A demethylation^44^. Interestingly, FTO was also found to be highly expressed in the positive samples (Fig. 5e), which was in accordance with the significant decrease in the abundance of the m^6^A peaks. Significantly, FTO expression tended to decline with disease improvement and virus inhibition (Fig. 5f). It has been reported that m^6^A is involved in the replication of some viruses; for example, in Zika infection, depletion of FTO decreased the viral titer, ZIKV RNA expression, and envelope protein levels^45^. Depletion of FTO also decreased extracellular HCV RNA levels and infectious virion production^46^. Taken together, these data lead us to hypothesize that along with participating in the replication of other viruses such as HCV^45^ and ZIKV^46^, m^6^A may also be involved in SFTSV infection and replication and that FTO is likely to be a potential target for treatment.

**Fig. 6:**
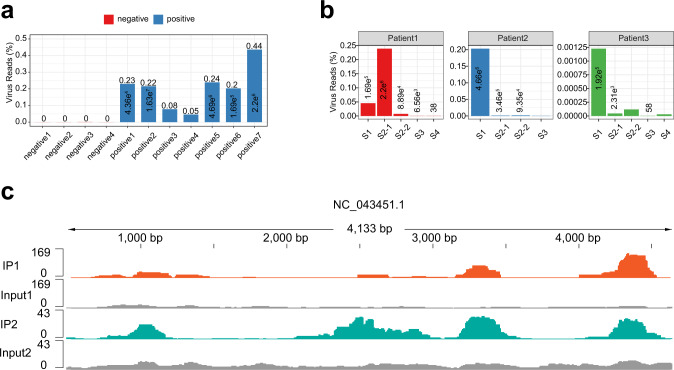
m^6^A modifications in SFTSV genome. **a**, **b** The percentage of SFTSV reads in the sequencing samples. The number above the column indicates the number of copies of the virus detected by qPCR of the clinical data. **c** Genome browser view of m^6^A-enriched regions in the SFTSV genome.

It was also reported that host methylation systems could modify the viral RNAs of several viruses, such as HIV, HBV, and flaviviruses including ZIKA virus, HCV, and dengue virus (DENV)^45,47–50^. Recent studies have shown that m^6^A in SARS-CoV-2 genomic RNA and negative-sense RNA in human and monkey cells are dynamically modified^51^. It is probable that the ability to imitate RNA methylation helps the viral RNA escape recognition by the host innate immune system. The sequencing data were mapped to the SFTSV genome, and it was found that the proportion of the reads number of the SFTSV genome was consistent with the trend of the SFTSV copy number measured in clinical tests, which again confirmed the reliability of our data (Fig. 6a, b). The analysis of m^6^A-seq illustrated that there were several potential m^6^A peaks in the SFTSV genome (Fig. 6c). Whether m^6^A in the SFTSV genome could regulate the replication or activity of the virus remains to be verified. Further studies may start with inhibitors of FTO and explore the relationship between m^6^A and SFTSV to provide new ideas for the treatment of SFTS.

## Discussion

Overall, the clinical laboratory features of SFTS patients such as platelet counts, AST levels, and viral loads were summarized. Our longitudinal sampling study suggests the need for monitoring of these clinical parameters throughout the treatments. In addition, transcriptomic and epigenetic characterizations were performed by RNA-seq and m^6^A-seq of white blood cells from SFTS patients. The differential gene expression revealed that at least two different factors could be associated with SFTSV-induced death: sharply decreased platelets and a delayed innate immune response. Moreover, the key m^6^A demethylation gene FTO exhibited a tendency of high expression after SFTSV infection, which was consistent with the significant decrease in the abundance of the m^6^A peaks observed in m^6^A-seq. The results of m^6^A-seq suggested that there could be a close relationship between the changes in m^6^A in the transcriptome and the changes in gene expression during SFTSV infection, suggesting an m^6^A-dependent gene regulation pathway. Potential m^6^A peaks in the SFTSV genome were also observed in the high-throughput sequencing results. By combining clinical test results and corresponding sequencing results, it was found that changes in myocardial enzymes, coagulation function, and inflammation may be useful to monitor the progression of SFTS disease and guide clinical treatment. Ostensibly, there have been very few studies of transcriptomic analysis on patients infected with SFTSV, and this work could well be the first epigenetic report. Starting with m^6^A-related enzymes, such as FTO, it will be of great significance to explore the influence of m^6^A on the replication and infection mechanism of SFTSV and provide new avenues for the treatment of SFTS. Our data and analysis provide a fresh perspective on the mechanisms underlying the pathogenicity of SFTSV and unravel the potential role of epigenetic modification of this virus with a high fatality rate.

There are still several limitations to the current study, such as the small number of clinical samples from a single center, and the lack of evaluation of the effects of antiviral therapy (i.e., ribavirin) on m^6^A modification. Ribavirin is a nucleotide analog with broad-spectrum antiviral activity and the Chinese Ministry of Health has approved ribavirin for SFTS treatment^52^. The proposed antiviral mechanisms include inosine monophosphate dehydrogenase inhibition, immunomodulatory effects, interference with RNA capping, polymerase inhibition, and lethal mutagenesis. In addition, ribavirin antiviral therapy can induce epigenetic reprogramming, mainly involving mainly changes in DNA methylation^53^ and histone modifications^54–57^. Although there is no report describing changes in m^6^A during ribavirin therapy, well-designed large-scale, multicentre clinical trials, and prospective studies are needed to determine whether anti-SFTSV treatment (i.e., ribavirin) affects the transcriptomic and epigenetic profile (i.e., m^6^A modification) of infected patients and to identify the underlying regulatory mechanisms.

## Methods

### Ethics statement

This study is in accordance with Chinese law. *Ethical Principles for Medical Research Involving Human Subjects* has been published to the public by the Decree No. 11 of National Health and Family Planning Commission of the People’s Republic of China, and came into force on December 1, 2016. The Medical Ethics Committee of Zhongnan Hospital of Wuhan University (Record number: EC-20200204-1004) has proved our study (approval number: 2019070). The ethics approval showed that the surgical removal of specimens must sign informed consents, and the clinical remaining blood samples are exempted from informed consent. Surgical removal of specimens was not part of the data presented in this study, but only the leftover blood samples. The explanation that exemption from informed consent is owing to the 6 facts that (1) this study used the EDTA anticoagulant blood remaining after the clinical laboratory completed the blood routine test (0.5–1 mL per sample); (2) this study did not directly contact the participants; (3) all participants had been discharged, and the research results are not required to tell the participants; (4) all results are only used for scientific exploration. It cannot be used for diagnosis or commercial usage; (5) all samples and relevant information were de-identified to protect the personal privacy; (6) this study did not generate additional risk to the participants.

### Some detailed information

The data report on conclusions is an observational study and the current study did not recruit patient specifically. The antiviral treatment is standard of care and not specifically part of this study. The two doctors (Zhigang Zhao and Shihui Song) in the author’s list contributed to the clinical information collection, results interpretation, and disease progression staging in this study. The remaining samples are those leftover blood after being used for clinical laboratory testing, and some of them are not enough for sequencing experiments. Each patient’s condition may develop differently, when and if there is a need for blood testing are totally depending on the therapy requirement of patients, so we do not have samples for each patient and each time point.

### Participants and samples

A total of 41 patients infected with SFTSV were recruited at the Zhongnan Hospital of Wuhan University between March 2020 and October 2020 (24 males and 17 females). The median age was 63.0 years (IQR: 59.5–69.0), and 58.5% of cases were males. All patients were diagnosed by the criteria for SFTS^58^ and confirmed by SFTSV nucleic acid testing. The clinical information was collected from the medical records of patients following a standard protocol during their clinical course. The baseline laboratory data referred to the results from the fresh blood samples obtained at admission. According to the Chinese management guideline for SFTS released in 2010, patients were received antiviral therapy (ribavirin) and support treatments. The clinical outcomes (such as recovered, deceased) were followed up to November 20, 2020.

Although we collected clinical data from 41 patients, not all remaining samples of these patients could be collected. The samples used in this study were all from the remaining specimens after clinical test, so we obtained informed consent exemptions approved by the ethics committee. The patient selection criteria are those who were diagnosed as SFTS and tested positive for the SFTSV using qRT-PCR. 7 SFTSV-positive specimens and 4 SFTSV-negative specimens from the SFTS patients were used for RNA-seq analysis. Blood samples from 4 patients were collected for m^6^A-seq. Due to the limited amount of nucleic acid extracted from a single sample, the positive sample libraries from the same patient were pooled for m^6^A-seq. The healthy group refers to the blood samples collected from the 21 healthy donors for physical examination. Since the RNA extracted from the blood samples of a single person is insufficient to perform m^6^A-seq, the blood samples of every seven healthy donors are mixed together as a control.

### Clinical laboratory tests

Serum SFTSV viral copy numbers were assayed by commercial TaqMan kits (Daan, Guangzhou, China). Liver function tests (serum alanine transaminase [ALT], aspartate transaminase [AST], total bilirubin [TBIL]), renal function tests (blood urea nitrogen [BUN] and creatinine), and C reactive protein [CRP] were determined with the AU5800 Chemistry Analyzer (Beckman, USA). Indicators of coagulation function (prothrombin time [PT], activated partial thromboplastin time [APTT]) were assayed by the ACLTOP700 coagulation analyzer (Werfen, Spain). The blood cell counts (white blood cell [WBC], neutrophil, lymphocyte, red blood cell [RBC], platelet), and hemoglobin were examined by automatic CELL-DYN1600 analyzer (Abbott, USA).

### Statistical analysis

The SPSS software, version 21.0 (IBM Inc., Chicago, IL) was used to analyze the statistical data. Mann–Whitney *U* test or *χ*² test was used to assess difference between groups. The results were presented as median (interquartile range). Pairwise deletion method was used to deal with missing data. All the *p*-values were two-sided and statistical significance level was at *α* = 0.05.

### RNA extraction

RNA was extracted from white blood cells using Direct-zol RNA Miniprep Kits (Zymo Research, R2050). The eluted product was incubated with DNase I (ThermoFisher Scientific, RNase-free, EN0521) at 37 °C for 30 min to further remove the DNA and then fragmented with RNA Fragmentation Reagents (Invitrogen, AM8740) at 70 °C for 15 min. Finally, the RNA was purified with RNA Clean and Concentrator^TM^-5 kits.

### Library preparation

The RNA without enrichment and the m^6^A-enriched RNA were used for library preparation performed with SMARTer® Stranded Total RNA-Seq Kit v2 (Takara Bio USA, Inc., 634411). Purification of the RNA-Seq library using VAHTS Clean Beads (Nanjing, China). All of the quality-ensured libraries were sequenced on the Illumina hiseq Xten platform in 150 bp paired-end mode (sequenced by Genewiz).

### Measuring differential gene expression levels using RT-qPCR

The detailed reaction conditions for reverse transcription assay are as follows: Reverse transcription reaction was prepared with total RNA, 0.5 µL random 6 mer primer (100 µM), 0.5 µL oligo dT (50 µM), dNTP mix (2.5 mM), and ddH_2_O to give a final volume of 10 μL. The mixture was incubated at 65 °C for 5 min and placed on ice for 2 min. Then 2 µL 10 × M-MuLV reverse transcriptase buffer, 1 µL M-MuLV reverse transcriptase, 0.3 µL RNase Inhibitor, 6.7 µL RNase-free H_2_O were added into the chilled mixture. The mixture was then subjected to the following thermal cycle using a T100™ Thermal Cycler (Bio-Rad): 25 °C for 15 min, 42 °C for 60 min, then heated at 65 °C for 20 min to inactivate the enzyme. The protocol of qPCR assay was described as follows: 1 µL RT product as template, 1 µL reverse primer (10 µM), and 1 µL RT primer (10 µM) was added into 2 × Hieff^®^ qPCR SYBR^®^ Green Master Mix (YEASEN) with a total volume of 20 µL. Then the mixture was performed the following thermal cycle: 95 °C for 5 min, 40 cycles of (95 °C for 10 s, 60 °C for 30 s, 72 °C for 20 s) using a CFX-96 Real-Time System (Bio-Rad, USA). The sequences of PCR primers that were used for qPCR were listed in Supplementary Table 2.

### m^6^A-seq and RNA-seq analysis

The human hg38 genome and list of transcripts v31 were downloaded from Gencode. The SFTSV virus genome was downloaded from NCBI (GenBank Accession GCF_003087855.1). Raw FASTQ reads were trimmed to remove adaptor contamination and aligned to the human reference genome using cutadapt^59^ and STAR 2.7.5a^60^, respectively. Reads <25 in length were removed, and only the proper pair and uniquely mapped alignments was persisted for the downstream pipelines. Then aligned reads were used for peak calling and enriched regions comparing by exomePeak. MetaPlotR package^61^ was used for creating metagene plots. HOMER was used to detect the sequence motif. Statistical analysis of the m^6^A peak in each transcript region was done. If the FDR < 0.05 and log_2_FC ≥ 1, the differentially expressed peak was considered as positive. For RNA-seq, differential expression genes were identified using three significance tests (exact test, likelihood ratio test, and quasi-likelihood F-test) in the edgeR package in R^47,62–64^. Genes were classified as differentially expressed between SFTS patients and recovery stage if *p* value < 0.001 and log_2_FC ≥ 1 for all three aforementioned tests. Counts for differentiating genes were then converted to counts per million (CPM) for each sample, and z-scores were calculated for each gene. Hierarchical clustering was performed using Euclidean distance. DAVID was used for Gene Ontology (GO) enrichment analysis.

## Supplementary information

Supplementary Information

nr-reporting-summary

## Data Availability

The data that support this study are available from the corresponding authors upon reasonable request. The sequencing data generated in this study have been deposited into the Gene Expression Omnibus (GEO) under the accession number GSE165518. Source data are provided with this paper.

## Source data

Source Data

## Code availability

The analysis scripts have been deposited into GitHub [http://github.com/sqhan-whu/SFTSV].
