# Supplementary material for: A longitudinal sampling study of transcriptomic and epigenetic profiles in patients with thrombocytopenia syndrome
Source: Nat Commun. 2021 Sep 24;12:5629. doi: 10.1038/s41467-021-25804-z (PMC8463551; doi:10.1038/s41467-021-25804-z)
Supplement: Supplementary file 1 — Supplementary Information [file 41467_2021_25804_MOESM1_ESM.pdf]

Supplementary Information

A longitudinal sampling study of transcriptomic and epigenetic profiles in patients with thrombocytopenia syndrome

Yafen Wang[1,6], Shaoqing Han[1,6], Ruoxi Ran[2,6], Anling Li[2,6], Huanyu Liu[3], Mingjun Liu[2], Yongwei Duan[2], Xiong Zhang[1], Zhigang Zhao[4], Shihui Song[5], Xiaocheng Weng[1,*], Songmei Liu[2,*], Xiang Zhou[1,*]

[1]College of Chemistry and Molecular Sciences, Wuhan University, Wuhan 430072, China

[2]Department of Clinical Laboratory, Center for Gene Diagnosis, and Program of Clinical Laboratory Medicine, Zhongnan Hospital of Wuhan University, Wuhan 430071, China

[3]Department of Obstetrics and Gynecology, Reproductive Medicine Center, Zhongnan Hospital of Wuhan University, Wuhan 430071, China

[4]Department of Emergency, Zhongnan Hospital of Wuhan University, Wuhan University, Wuhan 430071, China

[5]Department of Infectious Disease, Zhongnan Hospital of Wuhan University, Wuhan University, Wuhan 430071, China

[6]These authors contributed equally: Yafen Wang, Shaoqing Han, Ruoxi Ran, Anling Li

*Corresponding Authors.

Email: xcweng@whu.edu.cn; smliu@whu.edu.cn; xzhou@whu.edu.cn

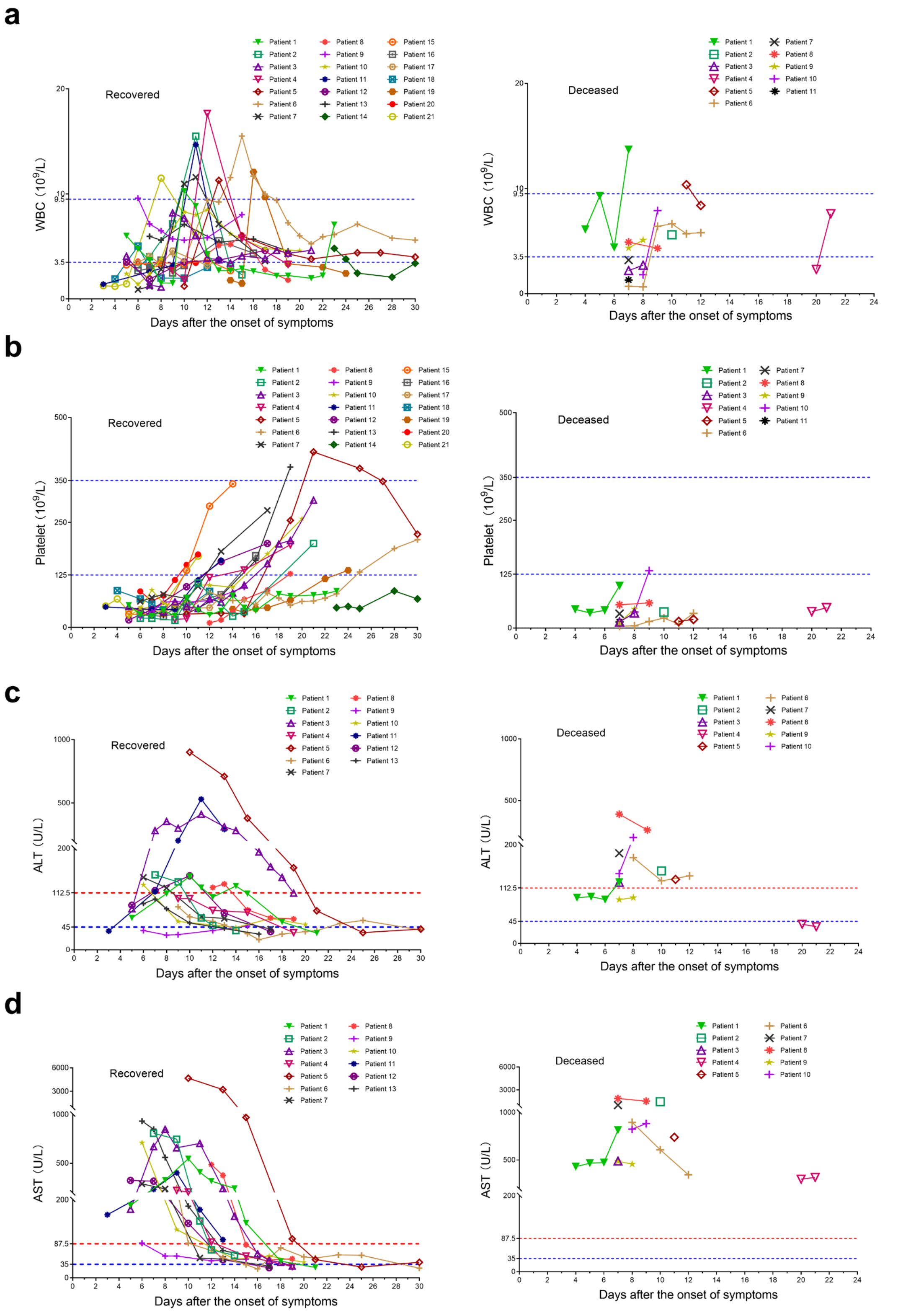

Supplementary Figure 1. Chronological changes of laboratory findings in patients with SFTSV infection. The chronological changes of WBC counts (**a**), platelet counts (**b**), serum ALT (**c**) and AST (**d**) are shown in the left panel and the right panel,

respectively. The dashed blue lines represent the clinical normal ranges of WBC (3.5 - 9.5 × 10^9/L), platelet (125 - 350 × 10^9/L), ALT (0 - 45 U/L), AST (0 - 35 U/L). The dashed red lines represent 2.5-fold of the up limit of the normal ranges for ALT and AST, which indicates requiring supportive treatment for liver function.

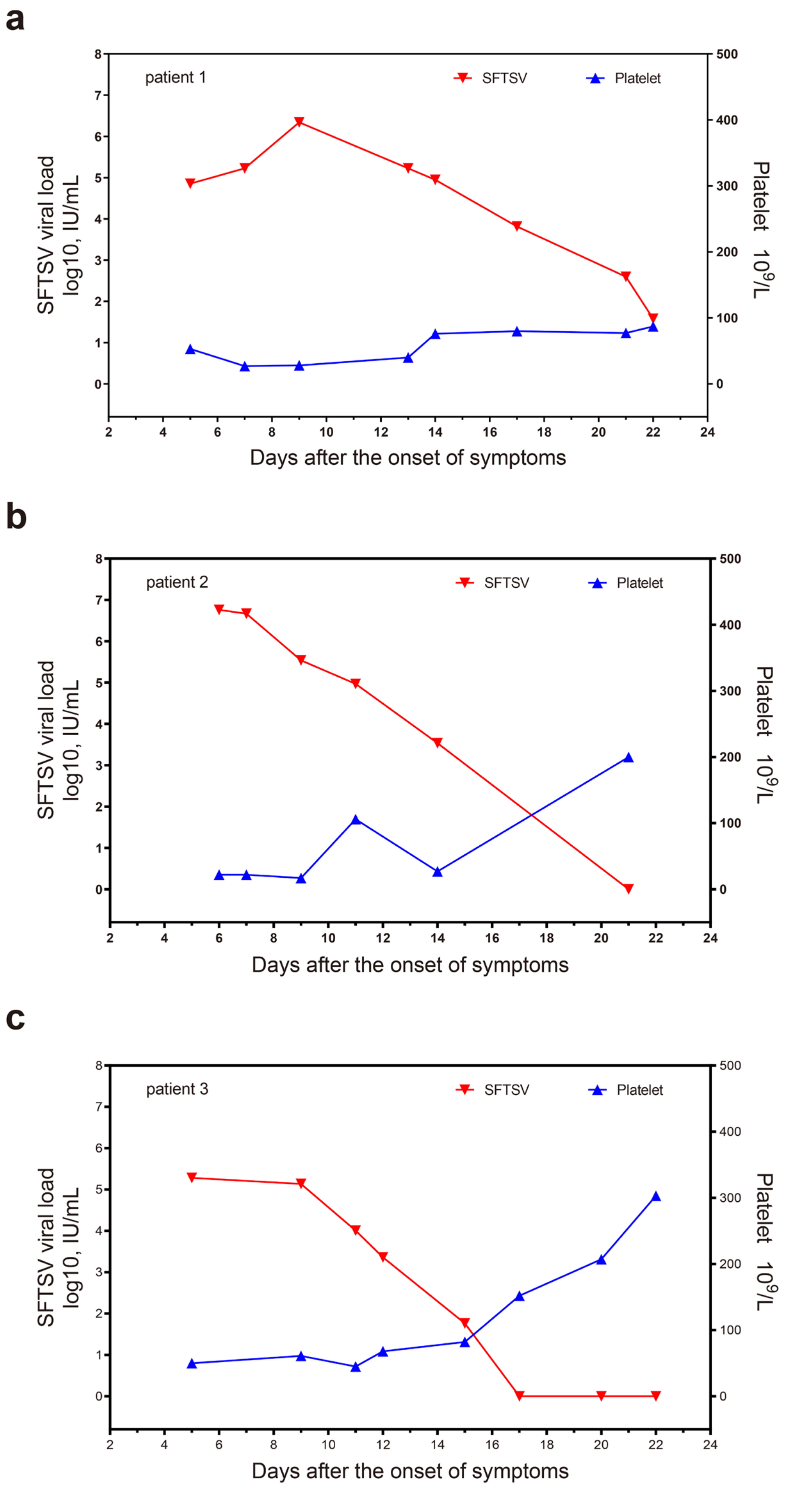

Supplementary Figure 2. The change pattern of the viral load of SFTSV and platelet counts in 3 representative patients at the same day. Each point represents a testing result of SFTSV and platelet at the same day. a, **b**, and **c** represent three different patients, respectively.

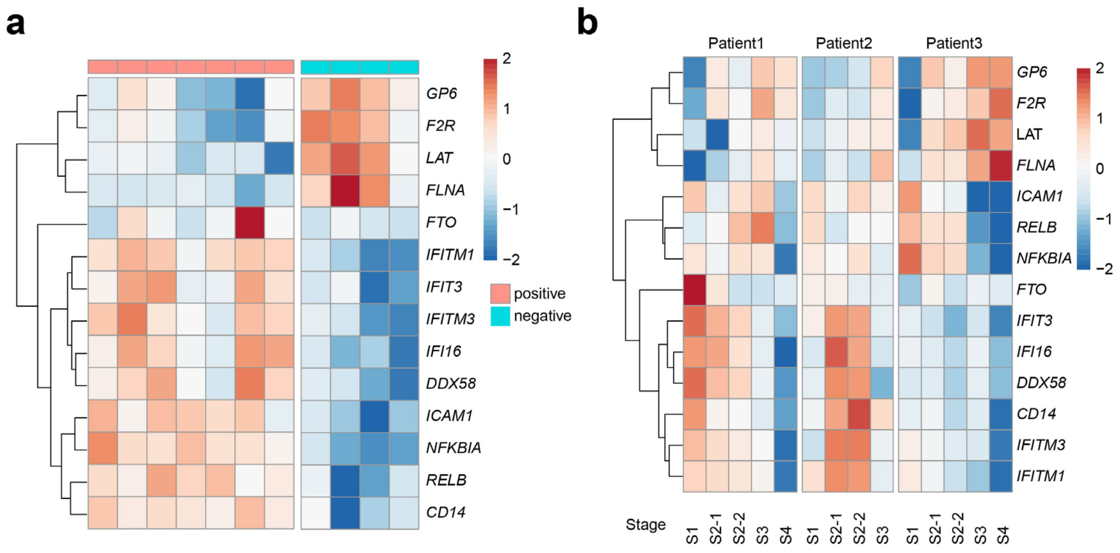

Supplementary Figure 3. The heat map of some differential genes identified by RNA-seq. a. Heat map of the 14 differential genes from 7 positive and 4 negative samples. **b.** Heat map of the 14 differential genes from the 3 patients with covering different stages of infection.

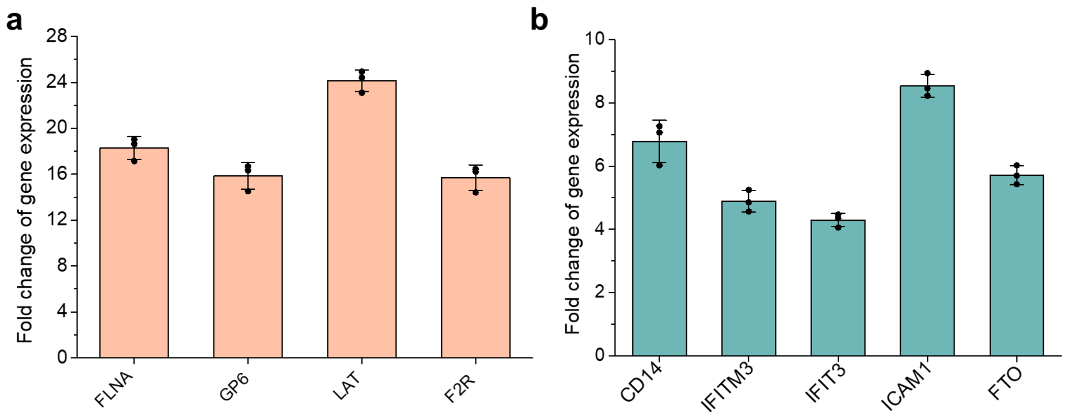

Supplementary Figure 4. qPCR analysis for validation some differential genes identified by RNA-seq. a. The differential genes up-regulated in the positive samples compared with the negative samples. **b.** The differential genes down-regulated in the positive samples compared with the negative samples (The vertical axis is the fold of these genes are expressed in the negative sample compared to the positive sample). (n = 3 independent experiments, data are represented as mean values ± SD).

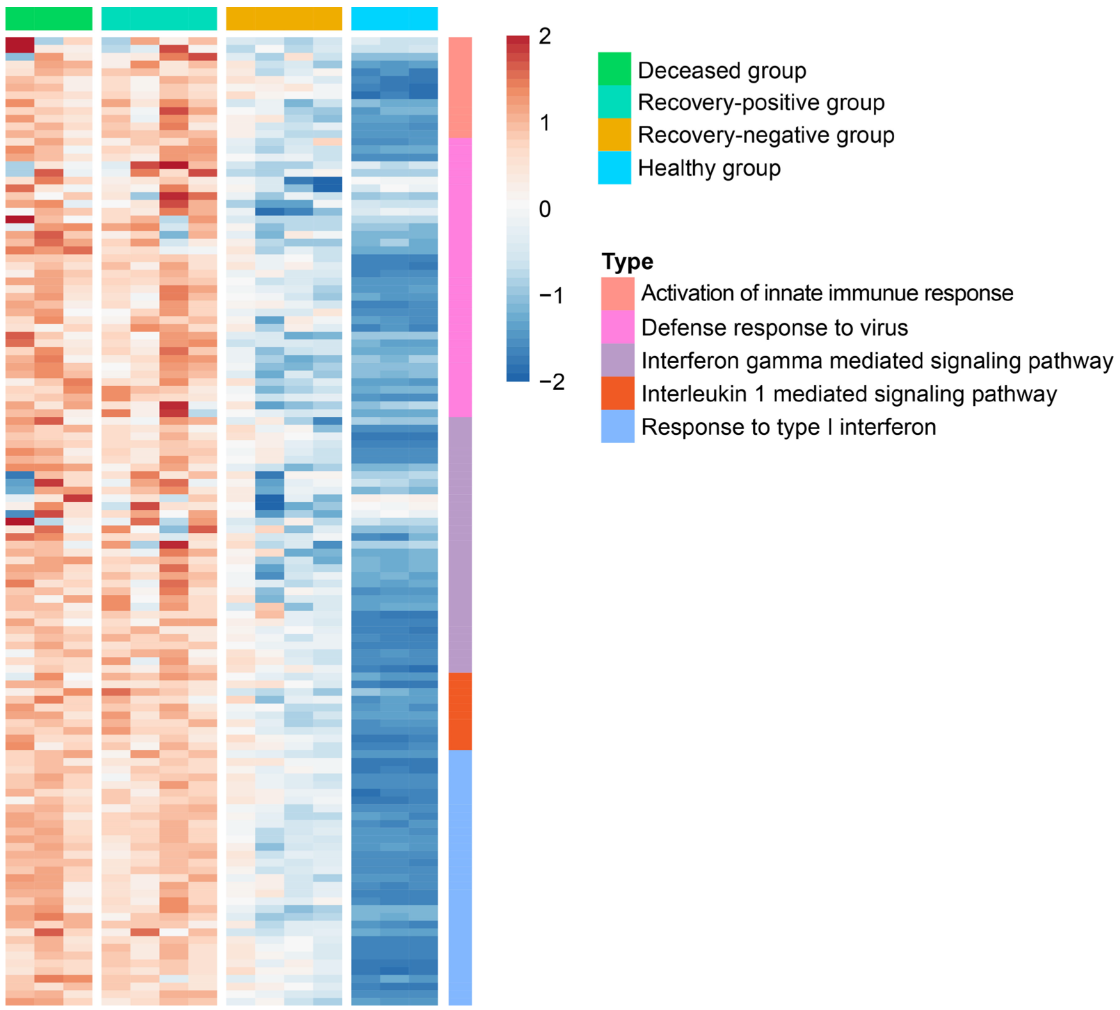

Supplementary Figure 5. Clustered heat map showing significant differential expression of 125 genes identified in the clinical outcomes. The 125 differentially expressed genes were identified across the deceased group, recovery-positive group (positive samples from recovering patients), recovery-negative group (negative samples from recovering patients) and healthy group.

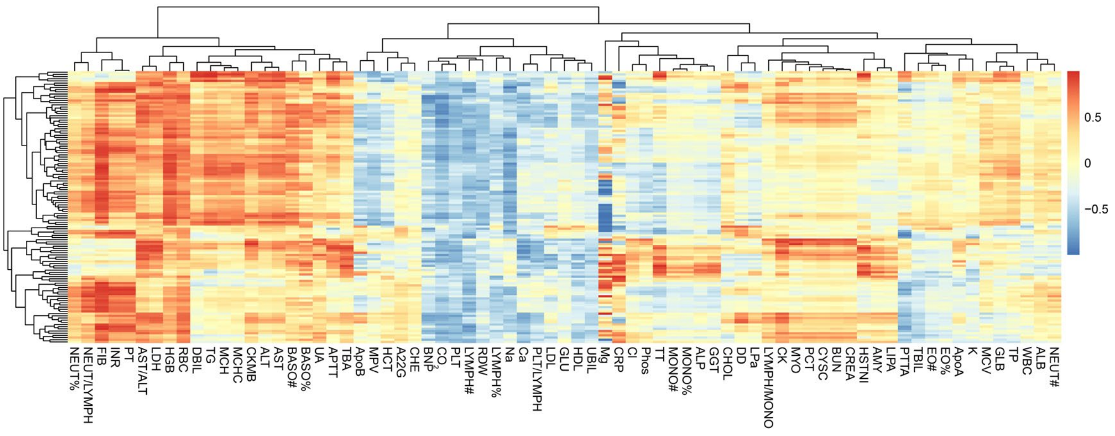

Supplementary Figure 6. Correlation between clinical markers and differential

genes. The heat maps of clinical data and sequencing data were analyzed using *Pearson* correlation analysis. The symbol of % represents the percentage, and the symbol of # represents the absolute value.

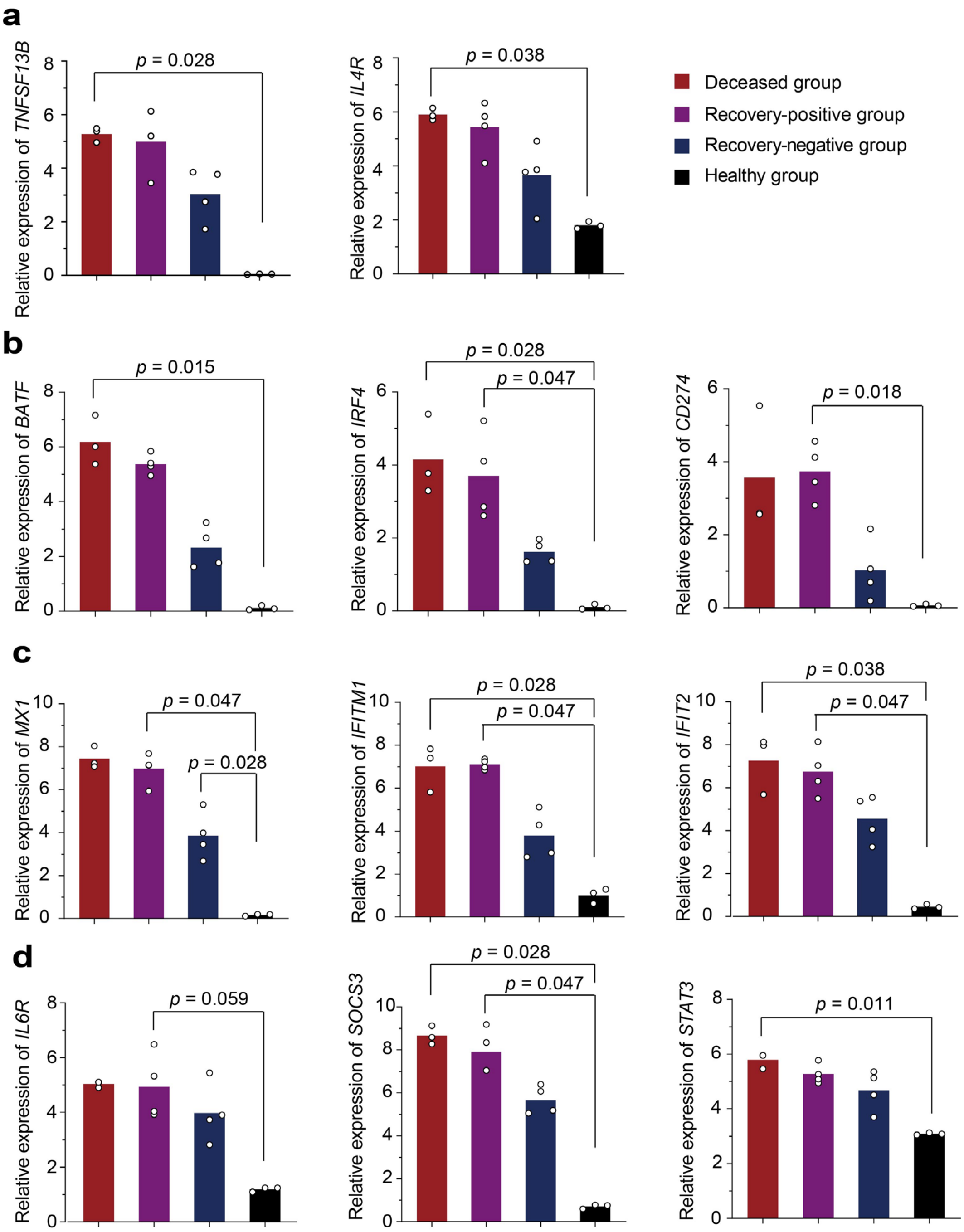

Supplementary Figure 7. Expression of genes related to immune and inflammatory cytokines. a. B cell activation (*TNFSF13B*, *IL4R*). **b.** T cell exhaustion (*BATF*, *IRF4*, *CD274*). **c.** Type I interferon (*MX1*, *IFITM1*, *IFIT2*). **d.** IL-6 (*IL6R*, *SOCS3*, *STAT3*). Deceased group (n = 3), Recovery-positive group (n = 4), Recovery-negative group (n = 4), and Healthy group (n = 3). Data indicate median. Each dot

represents a single patient. P values were determined by the two-sided Kruskal-Wallis test, followed by Dunn's post-test for multiple comparisons with median reported.

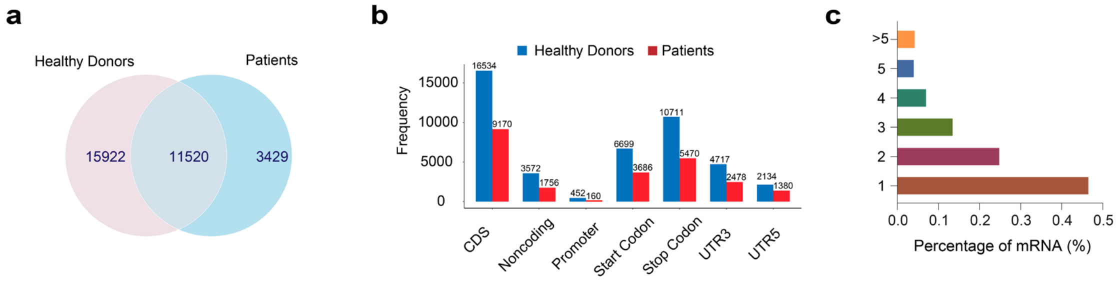

Supplementary Figure 8. Brief information of m^{6}A peaks. a. 77% of m^{6}A peaks in the SFTSV-infection samples are overlapping with the healthy controls' peaks. **b.** Percentage of mRNAs with different numbers of m^{6}A peaks. **c.** A histogram depicting the distribution of m^{6}A peaks in different transcript segments in SFTSV-positive samples and healthy donors' samples.

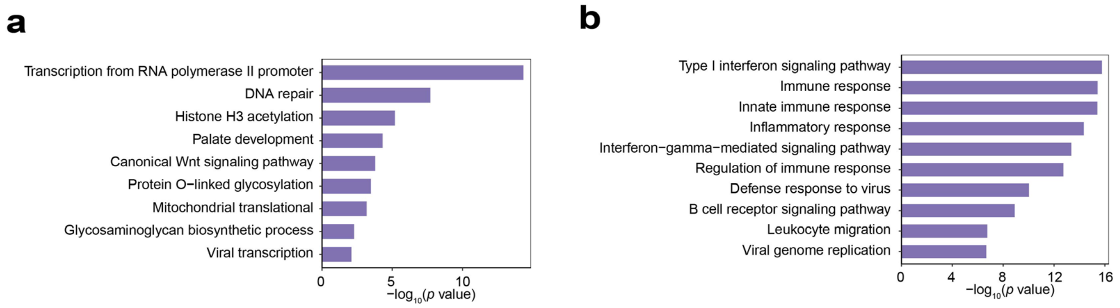

Supplementary Figure 9. GO analysis of enriched terms in biological processes associated with the disease of SFTSV. a. The main enriched terms of genes with downregulated m^{6}A abundance (the p value is provided by DAVID online tool). **b.** The main enriched terms of genes with upregulated m^{6}A abundance (the p value is provided by DAVID online tool).

Supplementary Table 1. Detailed information of the patients in Figure 2.

SFTSV	Samples	Days after the onset of symptoms	Stage
positive	positive1	6	Fever stage
	positive2	9	Deterioration/ Organ failure
	positive3	7	Fever stage
	positive4	9	Deterioration/ Organ failure
	positive5	9	Deterioration/ Organ failure
	positive6	9	Deterioration/ Organ failure
	positive7	8	Deterioration/ Organ failure
negative	negative1	22	Convalescence
	negative2	23	Convalescence
	negative3	19	Improving
	negative4	22	Convalescence

Supplementary Table 2. PCR primers that were used for qPCR

No	Gene name	Strand	Sequence
1	18S	F	AGGGAATTCCCGAGTAAGTGCG
		R	GCCTCACTAAACCATCCAA
2	CD14	F	CTGGAACAGGTGCCTAAAGGAC
		R	GTCCAGTGTCAGGTTATCCACC
3	IFITM3	F	CTGGGCTTCATAGCATTCGCCT
		R	AGATGTTCAGGCACTTGGCGGT
4	IFIT3	F	CCTGGAATGCTTACGGCAAGCT
		R	GAGCATCTGAGAGTCTGCCCAA
5	ICAM1	F	AGCGGCTGACGTGTGCAGTAAT
		R	TCTGAGACCTCTGGCTTCGTCA
6	FTO	F	CCAGAACCTGAGGAGAGAATGG
		R	CGATGTCTGTGAGGTCAAACGG
7	FLNA	F	CAACAAGTTCACTGTGGAGACC A
		R	TGTAGGTGCCAGCCTCATAAGG
8	GP6	F	CGCTGAACTGACCGTCTCATTC
		R	CAGGTTGCCCTTGGTGTAGTAC
9	LAT	F	ATCCTGGAGCGGCTAAGACTGA
		R	GTTCAGCTCCTGCAGATTCTCG
10	F2R	F	GTTTCTGGCTGTGGTGTATCCC
		R	CCTGGATGGTTTGCTCCTTGAG
